# PD0325901 alleviates thrombin-inhibited osteogenic differentiation through an IL-1β-activated feedback loop between MEK-Erk1/2 and NF-κB signal pathways: insights from bioinformatics and experimental verification

**DOI:** 10.3389/fimmu.2026.1730337

**Published:** 2026-03-09

**Authors:** Yang-Shuo Ge, Chun-Meng Huang, Jun Shen, Ting-Ting Meng, Min-Jun Zhao, Jian-Li Yin, Xin-Hui Huang, Liao-Lin Chen, Jia-Hui Luo, Yu-Qing Zhai, Jia-Wei Du, Yi-Lin Wang, Xue-Zong Wang, Ping-Ping Sun, Dao-Fang Ding

**Affiliations:** 1 Department of Rehabilitation Therapy, The Second Rehabilitation Hospital of Shanghai, Shanghai, China; 2 Institute of Rehabilitation Medicine, Shanghai Academy of Traditional Chinese Medicine, Shanghai, China; 3 Department of Orthopedic, Guanghua Hospital of Integrative Chinese and Western Medicine, Shanghai, China; 4 Arthritis Institute of Integrated Traditional Chinese and Western Medicine, Shanghai Academy of Traditional Chinese Medicine, Shanghai University of Traditional Chinese Medicine, Shanghai, China; 5 Shi’s Center of Orthopedics and Traumatology, Shuguang Hospital Affiliated to Shanghai University of Traditional Chinese Medicine, Shanghai, China

**Keywords:** MEK-ERK1/2, NF-κB, osteoblasts, osteoporosis, thrombin

## Abstract

**Objective:**

To elucidate the molecular mechanisms underlying thrombin-induced suppression of osteoblast differentiation, and to identify the MEK inhibitor PD0325901 (PD03) as a potential therapeutic candidate.

**Methods:**

Following treatment of primary rat osteoblasts with thrombin (20 U/mL) and PD03 (0.1 μM) during osteogenic induction, the cells were harvested and subjected to RNA sequencing to identify differentially expressed genes (DEGs). The combination of network pharmacology and RNA sequencing was used to predict the targets of PD03 in thrombin-induced osteoblasts. Alkaline phosphatase (ALP) activity was assessed through staining and quantitative analysis; the expression of osteogenic genes was measured by quantitative PCR (qPCR) and western blot; mineralized nodule formation was evaluated by Alizarin Red S staining; the expression of signaling pathway-related proteins (p-Erk1/2, p-Stat3, p-p65, MMP-9, COX-2) and proliferation-related proteins (PCNA and MCM2) were examined by western blot; nuclear localization of NF-κB was observed by immunofluorescence (IF); intracellular calcium levels were quantified using a calcium assay and probe labeling; osteoblast proliferation was evaluated by EdU staining; IL-1β secretion in cell supernatants was detected by ELISA; the expression of IL-1RA was measured by western blot; the effects of MMP-9 knockdown and COX-2 overexpression on osteogenic differentiation were investigated.

**Results:**

Thrombin promoted osteoblast proliferation and inhibited osteogenic differentiation by upregulating inflammatory factors and activating inflammatory signaling pathways, including MEK-Erk1/2 and NF-κB, which in turn reduced ALP activity, calcium ion influx, expression of osteogenic markers (e.g., Col1α1, Runx2, OCN), and mineralized nodule formation. PD03 reverses these effects by suppressing thrombin-induced activation of IL-1β-dependent signaling pathway, in which the downstream gene MMP-9 plays a critical role.

**Conclusion:**

PD03 inhibits thrombin-induced activation of the IL-1β-mediated feedback loop between the MEK-Erk1/2 and NF-κB pathways, thereby restoring bone formation and offering a promising therapeutic approach for mitigating bone loss in patients with elevated thrombin levels.

## 1 Introduction

Osteoporosis (OP) is a systemic skeletal disease characterized by low bone mass, deterioration of bone microarchitecture, increased bone fragility, and a high risk of fractures and disability (1). In clinical practice, OP patients often experience pain, hunchback, and fractures, which seriously affect their quality of life. According to its etiology, OP can be classified into primary OP and secondary OP (2). The global overall prevalence of OP is 18.3%, with a prevalence of 23.1% in women and 11.7% in men (3). As the global population ages, the number of individuals affected by OP is expected to increase annually, drawing growing attention from researchers.

Currently, the primary clinical medication for OP primarily targets the activity of osteoclasts (4, 5). However, bone homeostasis is maintained through a dynamic balance between osteoclast-mediated bone resorption and osteoblast-mediated bone formation. Disruption of this balance leads to the development of OP. Studies have demonstrated that the systemic immune-inflammation index (SII) is negatively correlated with bone mineral density and positively correlated with the risk of OP, suggesting a direct association between inflammation and the development of OP (6, 7). Proinflammatory cytokines such as IL-1β and TNF-α are key regulators of bone resorption, promoting the differentiation of osteoclast precursors into mature osteoclasts by inducing the expression of RANKL (8). In addition to promoting bone resorption by stimulating osteoclast formation, these inflammatory cytokines also impair bone formation by directly inhibiting osteoblast differentiation. An inflammatory environment triggered by elevated cytokines, such as TNF-α, IL−1β, and IL-6, can suppress ALP activity and the expression of osteoblastic markers, thereby impairing osteoblast differentiation and mineral deposition. Inflammatory cytokines also compromise the regenerative potential of stem cells by activating pathways such as NF-κB and suppressing Wnt/β−catenin signaling, resulting in reduced osteoblast differentiation and impaired matrix mineralization (9). This contributes to disrupted bone homeostasis and potentially leads to inflammation-driven bone loss (10–12). Therefore, anti-inflammatory therapy has become a potential therapeutic strategy for OP.

The high risk of OP and fractures associated with hemophilia (PWH) is mainly related to the deficiency of coagulation factors VIII and IX. Furthermore, the lower thrombin generation in PWH may decrease the number of osteoblasts, thereby disrupting the RANK/RANKL/OPG pathway and leading to reduced bone formation (13, 14).

Thrombin plays a pivotal role in the pathological processes of thrombosis and fibrosis, serving as a critical mediator in the coagulation cascade (15). Recent studies have increasingly demonstrated that, beyond its essential function in hemostasis, thrombin also acts as a potent inflammatory mediator in a wide array of diseases (16, 17). The proinflammatory effects of thrombin are usually mediated through activating protease-activated receptor-1 (PAR-1) (16). Furthermore, thrombin exacerbates tissue damage by amplifying alveolar inflammation and promoting a fibrotic phenotype, particularly in patients with COVID-19-associated lung injury (18). Additionally, thrombin drives neuroinflammation by activating microglia, astrocytes, and endothelial cells, thereby promoting a proinflammatory response (19).

Moreover, hirudin, as the most potent natural thrombin inhibitor currently known, enhances the proliferation of human bone marrow mesenchymal stem cells (HBMSCs), increases ALP activity, promotes mineralized nodule formation (as shown by Alizarin Red S staining), and upregulates the expression of osteogenesis-related genes, thereby promoting osteogenic differentiation (20). These findings collectively suggest that thrombin may function as a proinflammatory mediator in inhibiting osteogenic differentiation. Subsequently, based on RNA sequencing analysis integrated with network pharmacology and molecular docking data, thrombin was predicted to inhibit osteoblast differentiation by activating key inflammatory pathways, including MEK-Erk1/2, NF-κB, and IL-6/Stat3 pathways. Further *in vitro* experiments demonstrated that thrombin induced the release of IL-1β, activating a feedback loop involving NF-κB and the MEK-Erk1/2 pathways, which in turn promoted the production of numerous inflammatory cytokines associated with the suppression of osteogenic differentiation. Ultimately, we found that PD03 reversed the inhibitory effects of thrombin on osteogenic differentiation by targeting the upstream molecule of the feedback loop, suggesting that PD03 may serve as a potential therapeutic agent for inflammation-induced bone loss.

## 2 Materials and methods

### 2.1 Osteoblast culture

Primary osteoblasts were isolated from the calvaria of 24-hour-old male Sprague–Dawley rats, which were provided by Shanghai Xipur-Biak Experimental Animal Co., Ltd. (Shanghai, China). Calvarial bones were digested in 0.1% collagenase type I (BS163, Biosharp, China) at 37 °C for 60 minutes in a shaking water bath. Isolated osteoblasts were maintained in high-glucose Dulbecco’s Modified Eagle’s Medium (DMEM) supplemented with 10% fetal bovine serum (10270-106, Thermo Fisher, USA) and penicillin–streptomycin (ST488, Beyotime, China). First-passage osteoblasts were used for functional analyses. To evaluate the levels of phosphorylated proteins after intervention with thrombin and inhibitors, osteoblasts were cultured in serum-free DMEM for five hours prior to treatment. Subsequently, serum-free DMEM supplemented with 20U/mL thrombin (ST1699, Beyotime, China), thrombin + 0.1 μM PD03 (21–23) (S1036, Selleck, China) were administered for 5, 15, and 30 minutes to detect the expression levels of p-Stat3 and p-Erk1/2, and for 15, 30, and 60 minutes to detect the expression level of p-NF-κB; thrombin + 5 μM C188-9 (S8605, Selleck, China) for 5, 15, and 30 minutes to detect p-Stat3 and p-Erk1/2; thrombin + 0.1 μM QNZ (S4902, Selleck, China) for 15, 30, and 60 minutes to detect p-Erk1/2; 20ng/mL IL-1β (501-RL, Bio-Techne, USA) + 0.1 μM PD03 were administered for 15, 30, and 60 minutes to detect p-NF-κB; IL-1β + 0.1 μM QNZ were administered for 15, 30, and 60 minutes to detect p-Erk1/2.

### 2.2 Osteogenic differentiation and experimental groups

Osteoblasts were seeded in 6-well plates and incubated until they reached 75% confluence. Then, the maintenance medium was replaced with an osteogenic induction medium, which contains 10% fetal bovine serum, 10 mM β-glycerophosphate, 50 μg/mL L-ascorbic acid, and 10^-7^ M dexamethasone, with medium changes every 2–3 days. Based on the experimental design, osteoblasts were divided into the following groups: control group (osteoblasts were cultured in osteogenic induction medium), thrombin group (osteoblasts were cultured in osteogenic induction medium  in the presence of  20 U/mL thrombin), PD03 group (osteoblasts were cultured in osteogenic induction medium  in the presence of  20 U/mL thrombin and 0.1 μM PD03), C188 group (osteoblasts were cultured in osteogenic induction medium in the presence of  20 U/mL thrombin  and  5µM C188-9), QNZ group (osteoblasts were cultured in osteogenic induction medium  in the presence of  20 U/mL thrombin  and  0.1µM QNZ) and Vorapaxar group [osteoblasts were cultured in osteogenic induction medium  in the presence of  20 U/mL thrombin  and  0.1 μM, 0.5 μM, or 1 μM Vorapaxar (HY-10119, MCE, USA)]. Osteogenic differentiation was induced for 7 days to evaluate the expression of osteogenic genes at both the protein and mRNA levels.

### 2.3 Transient transfection of primary osteoblasts using pCDH-GFP-COX-2 plasmid and MMP-9 siRNA

Primary osteoblasts were seeded into 6-well plates at 70-80% confluence and transfected using 10 μL Lipofectamine 2000 (11668019, Invitrogen) and 2.0 μg of the pCDH-GFP-COX-2 plasmid or MMP-9 siRNA (Sangon, China). Six hours post-transfection, the medium was optionally refreshed. The efficiency of COX-2 overexpression and MMP-9 knockdown was verified by western blot. For osteogenic analysis, induction medium was added 24 hours post-transfection, with mRNA levels of osteogenic genes, ALP staining and activities evaluated on day 7.

### 2.4 RNA-seq analysis and bioinformatics pipeline

Osteoblasts were induced with osteogenic differentiation medium for 7 days and RNA was collected from each group with three independent samples. Both the preparation of a cDNA library and sequencing were performed with an Illumina HiSeq 4000 device at Shanghai Life Genes Technology Co., Ltd. RNA quality was analyzed by Bioanalyzer 2100 (Agilent Technologies, CA, USA) and an RNA Nano 6000 Assay Kit. The sequencing library was constructed using the NEB Next Ultra RNA Library Prep Kit for Illumina (NEB, USA).

A comprehensive bioinformatics workflow was implemented for downstream analysis. Raw sequencing reads were first quality−trimmed and adapter−filtered using Fastp (v0.23.2), and the resulting clean reads were then aligned to the rat reference genome (Rattus norvegicus, Rnor_6.0) using HISAT2 (v2.2.1). Gene-level read counts were generated with featureCounts (v2.0.3).

Differential expression analysis was performed in R using the DESeq2 package (v1.38.3), with normalization and statistical testing based on a negative binomial generalized linear model. Genes were considered differentially expressed if they met both of the following criteria: an adjusted P-value (False Discovery Rate, FDR) < 0.05 and an absolute |log_2_FoldChange| > 0.58.

Finally, functional enrichment analysis of the differentially expressed genes was conducted using the clusterProfiler package (v4.6.2) for both Gene Ontology (GO) and Kyoto Encyclopedia of Genes and Genomes (KEGG) pathway terms, with significance defined as FDR < 0.05.

### 2.5 Network pharmacology analysis

PD03 was searched in the Pubchem [https://pubchem.ncbi.nlm.nih.gov/database and its SMILES was C1=CC(=C(C=C1I) F) NC2=C(C=CC(=C2F) F) C(=O) NOC[C H] (CO)O. The SMILES was entered into the SwissTargetPrediction [http://swisstargetprediction.ch//] database to predict PD03 targets and further screen targets with a probability threshold greater than 0.

OP-related genes, as well as protein targets, were identified using the Genecards database [https://www.genecards.org/] using the keyword “osteoporosis”. The potential targets of PD03 and OP-related target genes were imported into Venny 2.1 [https://bioinfogp.cnb.csic.es/tools/venny/] online tool, and the two sets were intersected to create custom Venny diagrams.

### 2.6 Construction of the PPI network

To explore the functional interactions between proteins, a PPI network was constructed using the DEGs identified from sequencing results, as well as the common targets of OP and PD03. Proteins with a node degree greater than 3 were selected to ensure both focus and comprehensiveness of the network. The targeted genes were imported into the string database (https://string-db.org) for PPI analysis, with the minimum required interaction score set at 0.9. The resulting PPI data were downloaded as a TSV file and imported into Cytoscape 3.10.1 for visualization.

### 2.7 GO and KEGG pathway enrichment analyses

To explore the potential biological functions of the identified targets, including DEGs and the common genes from OP and PD03, these genes were submitted to the Metascape database (https://metascape.org/) for Gene Ontology (GO) analysis, with the parameters set as Min Overlap = 3 and P-value Cutoff = 0.01. Meanwhile, Kyoto Encyclopedia of Genes and Genomes (KEGG) pathway analysis was performed using the DAVID database (https://davidbioinformatics.nih.gov/), obtaining information related to Cellular Component (CC), Molecular Function (MF), and Biological Process (BP). The top 15 GO terms and top 15 KEGG pathways based on P-values were selected for further analysis. The results were visualized using the bioinformatics online platform (http://www.bioinformatics.com.cn/).

### 2.8 Molecular docking analysis

To further validate the interaction between the core targets identified through network pharmacology analysis and the active compound(s), molecular docking was performed. The top 10 DEGs from the 50 overlapping targets among PD03-related, thrombin-related, and osteoporosis-related were chosen, and the binding capacities of PD03 with the candidate proteins were evaluated using molecular docking. The 3D structures of the top 10 target proteins were obtained from the Protein Data Bank (PDB)(https://www.rcsb.org/), and the structure of the compound was retrieved from the PubChem database (https://pubchem.ncbi.nlm.nih.gov). The initial ligands and water molecules were eliminated with PyMOL software (DeLano Scientific LLC, USA). Docking was performed using AutoDock Vina (version 1.5.7), and the binding affinity was evaluated based on the docking scores (binding free energy, kcal/mol).

### 2.9 EdU assay

After 24 h of treatment with thrombin and PD03, osteoblast proliferation was assessed using the BeyoClick™ EdU Kit (Cat. No. ST067, Beyotime, China), with nuclei counterstained by DAPI. After washing with PBS, fluorescent images were acquired using an inverted microscope (Olympus IX73, Tokyo, Japan). The percentage of Edu-positive osteoblasts was further analyzed using six different images with ImageJ software (NIH).

### 2.10 Assessment of ALP activities

Osteogenically differentiated cells were obtained after 7 days of induction in osteogenic medium. Protein concentrations were measured using the bicinchoninic acid (BCA) assay kit (Cat. no. 23227, Pierce, USA). For the ALP activity assay, 20 µg of total protein was mixed with 100 µL of pNPP substrate solution (1-Step™ PNPP, Cat. No. 37621, Pierce, USA) and incubated at room temperature for 30 minutes. The reaction was terminated by adding 50 µL of 2 M NaOH. The absorbance of each well was measured at 405 nm using a microplate reader.

### 2.11 Alizarin red S staining

After 21 days of osteogenic differentiation, the osteoblasts were fixed in 4% paraformaldehyde (PFA) at room temperature for 20 minutes. Subsequently, the cells were incubated with Alizarin Red S staining solution (Cat. No. ALIR-10001, Cyagen, China) for 20 minutes. After staining, the cells were washed thoroughly with PBS three times to visualize calcium deposition.

### 2.12 The calcium colorimetric assay kit

Calcium ion content was measured using a calcium colorimetric assay kit (Cat. No. S1063S, Beyotime, China). After 7 days of osteogenic differentiation, the culture medium was removed, and the cells were washed three times with PBS. Lysis buffer (200 μL per well) was then added to each well of a 6-well plate on ice. The lysates were centrifuged at 14,000 × g for 5 minutes at 4 °C. Equal amounts of protein were used for subsequent analysis. Absorbance at 575 nm was measured using a BioTek microplate reader. The calcium concentration was calculated based on the standard curve generated according to the manufacturer’s instructions.

### 2.13 Intracellular calcium measurement using Fluo-4 AM

After treatment described in 2.12, cells were loaded with 1 μM Fluo-4 AM dye working solution (Beyotime, S1060) at 37 °C for 30 minutes to allow for probe loading. After incubation, wash the cells three times with PBS and detect intracellular Ca²^+^ levels using a fluorescence microscope (Olympus IX73, Tokyo, Japan).

### 2.14 Cellular immunofluorescence

After 24 hours of treatment with thrombin, PD03, and QNZ, osteoblasts were washed twice with PBS for 5 minutes each time. Then they were fixed in 4% paraformaldehyde (PFA) in PBS for 15 minutes. Subsequently, they were permeabilized with 0.1% Triton X-100 in PBS for 10 minutes. Subsequently, cells were blocked with 5% BSA in PBS for 60 minutes in a humidified chamber. IL-1β (diluted 1:800, GB11113, Servicebio, China) were applied overnight at 4 °C. After washing with PBST, a fluorophore-conjugated secondary antibody (diluted 1:1000, A-21428, Thermofisher, USA) was incubated for 1 hour in the dark. Nuclei were counterstained with DAPI (1 µg/mL, Proteintech, CM07245) for 5 min. Fluorescent images were captured using an inverted microscope (Olympus IX73, Tokyo, Japan).

### 2.15 NF-κB nuclear translocation assay

Osteoblasts were serum-starved in DMEM for 5 hours prior to treatment and then incubated with serum-free DMEM containing 20 U/mL thrombin and 0.1 μM PD03 for 30 min. NF-κB nuclear translocation was subsequently assessed using the NF-κB Activation and Nuclear Translocation Assay Kit (SN371, Beyotime, China). After washing with PBS, fluorescence images were captured using an inverted microscope (Olympus IX73, Tokyo, Japan).

### 2.16 Western blot analysis

Osteoblasts were lysed by RIPA buffer (P0013B, Beyotime, China) containing phenylmethanesulfonyl fluoride (PMSF) (ST505, Beyotime, China). Following centrifugation at 4 °C for 10 minutes at 12,000 g, the concentrations of the lysates were analyzed using a bicinchoninic acid (BCA) kit (Cat. No. 23227, Pierce, USA). The samples were separated and transferred to membranes. The membrane was probed with primary antibodies overnight at 4 °C and then stained with Anti-rabbit HRP-linked antibody (7074P2, CST, USA) and Anti-mouse HRP-linked antibody (7076P2, CST, USA). Protein bands were detected using enhanced chemiluminescence (Pierce Biotechnology, Rockford, USA). The following antibodies were used in this study: β-actin (AF5001, Beyotime, China), Col1α1 (ab270993, Abcam, UK), Runx2 (AF2593, Beyotime, China), OCN (AF6297, Beyotime, China), Igf1 (AF7179, Beyotime, China), Wnt5a (29793-1-AP, Proteintech, USA), Tgfb3 (AF8142, Beyotime, China), Spp1 (A5427, Bimake, USA), COX-2 (F0327, Bimake, USA), Matrix Metalloproteinase-9 (MMP-9, AF5234, Beyotime, China), Minichromosome Maintenance Complex Component 2 (MCM2, A5172, Bimake, USA), Proliferating Cell Nuclear Antigen (PCNA, SC-25280, Santa, USA), PAR-1 (AF6837, Beyotime, China), IL-1RA (AF7218, Beyotime, China), p65 (8242S, CST, USA), p-p65 (3033T, CST, USA), Erk1/2 (4695T, CST, USA), p-Erk1/2 (4370S, CST, USA), p-Stat3 (Y705) (9145S, CST, USA), p-Stat3 (S727) (9134S, CST, USA), and Stat3 (4904T, CST, USA).

### 2.17 RNA extraction, reverse transcription, and qRT-PCR

RNA was isolated from osteoblasts with a RNeasy Kit (R0027, Beyotime, China), and reverse-transcribed into cDNA with an RT Reagent Kit (AG11706, AG, China). qPCR was then performed using SYBR qPCR Master Mix (AG11718, AG, China). The 2^-ΔΔCt^ method was used to measure the relative expression of target genes. **Table 1** lists the primers used, and the samples were examined in triplicate.

**Table 1:** The primer sequences used in the study.

Gene name	mRNA sequences (5’-3’)
β-actin-F	AGATCAAGATCATTGCTCCTCCTG
β-actin-R	GGGTGTAAAACGCAGCTCAG
Col1α1-F	CATGTTCAGCTTTGTGGACCT
Col1α1-R	GCAGCTGACTTCAGGGATGT
Runx2-F	AAGGAGCACAAACATGGCTG
Runx2-R	TCTTAGGGTCTCGGAGGGAA
Osterix-F	CAAGGGTTAGGTGGTGGGC
Osterix-R	TCTTGGGGTAGGACATGCTG
OCN-F	CCCAATTGTGACGAGCTAGC
OCN-R	CTGTGCCGTCCATACTTTCG
OPG-F	TGAGGTTTCCAGAGGACCAC
OPG-R	GGAAAGGTTTCCTGGGTTGT
IL-6-F	CCACTGCCTTCCCTACTTCA
IL-6-R	TTCTGACAGTGCATCATCGC
TNF-α-F	CGTCGTAGCAAACCACCAAG
TNF-α-R	GAGGCTGACTTTCTCCTGGT
Lif-F	CAGTGCCAATGCCCTCTTTA
Lif-R	GCATGGAAAGGTGGGAAATC
IL-1β-F	GCCCATCCTCTGTGACTCAT
IL-1β-R	AGGCCACAGGTATTTTGTCG
Cxcl1-F	GGCTGGGATTCACCTCAAGAA
Cxcl1-R	TGTGGCTATGACTTCGGTTTG
Cxcl3-F	ACCAGCCTTCAGGGACTGT
Cxcl3-R	GGCTATGACTTCTGTCTGGGT
Cxcl6-F	TTCTGCTGCTGTTCACACTG
Cxcl6-R	TATCAACGGAGCTTGTGGGT
Cxcl16-F	AGTGGGTCCGTGAACTAGTG
Cxcl16-R	GAAAAGTGGACTGCTGGGTG
IL-33-F	TGAAGAAAGGCCAACAGAG
IL-33-R	AGCAGAGAAAAGGAAAGGG

### 2.18 Enzyme-linked immunosorbent assay

After 48 hours of treatment with thrombin, PD03, and QNZ, the cell supernatant was collected. IL-1β concentrations in culture supernatants were measured in duplicate using the Rat IL-1β ELISA KIT (JHN85275, Jinhengnuo Biotechnology, China), following the manufacturer’s instructions. Absorbance at 450 nm was recorded, and the concentrations were calculated according to the instruction.

### 2.19 Statistical analysis

All quantitative experiments, including qRT-PCR, western blot, ALP activity, calcium assay, EdU analysis, were performed using independent biological replicates and technical replicates to ensure reliability and generalizability (n ≥ 3). Data from technical replicates per biological replicate were averaged to obtain a single value for statistical analysis. For morphological or observational assays (e.g., immunofluorescence, ALP, and Alizarin Red S staining), representative images are shown from at least three independent experiments, with multiple randomly selected fields analyzed.

Data were analyzed using SPSS v19.0 (IBM, USA) and GraphPad Prism 9.0 (GraphPad Software, USA) and are presented as mean ± standard deviation (SD). For comparisons across multiple groups, one-way analysis of variance (ANOVA) was performed to assess overall significance. When ANOVA indicated a significant difference, Tukey’s *post-hoc* test was applied for all pairwise comparisons to control the family-wise error rate. For comparisons between two groups, Student’s t-test was used. A P-value ≤ 0.05 was considered statistically significant.

## 3 Results

### 3.1 PD03 reverses thrombin-mediated inhibition of osteoblastic differentiation

To determine the appropriate concentrations of thrombin required for subsequent experiments, we examined the effects of various concentrations (0, 5, 10, 20, 50 U/mL) on the ALP activity of osteoblasts. Thrombin at a concentration of 20 U/mL exhibited a significant inhibitory effect on ALP activity (**Supplementary Figure 1****),** and was selected as the concentration for subsequent studies. For PD03, we referred to established literature indicating that 0.1 μM effectively inhibits MEK–Erk1/2 signaling in various cell types without causing cytotoxicity (21–23). The intervention with 20 U/mL thrombin and 0.1μM PD03 showed no obvious effects on cell growth or viability, as assessed by cell density and morphology (**Supplementary Figure 2**).

**Figure 1:**
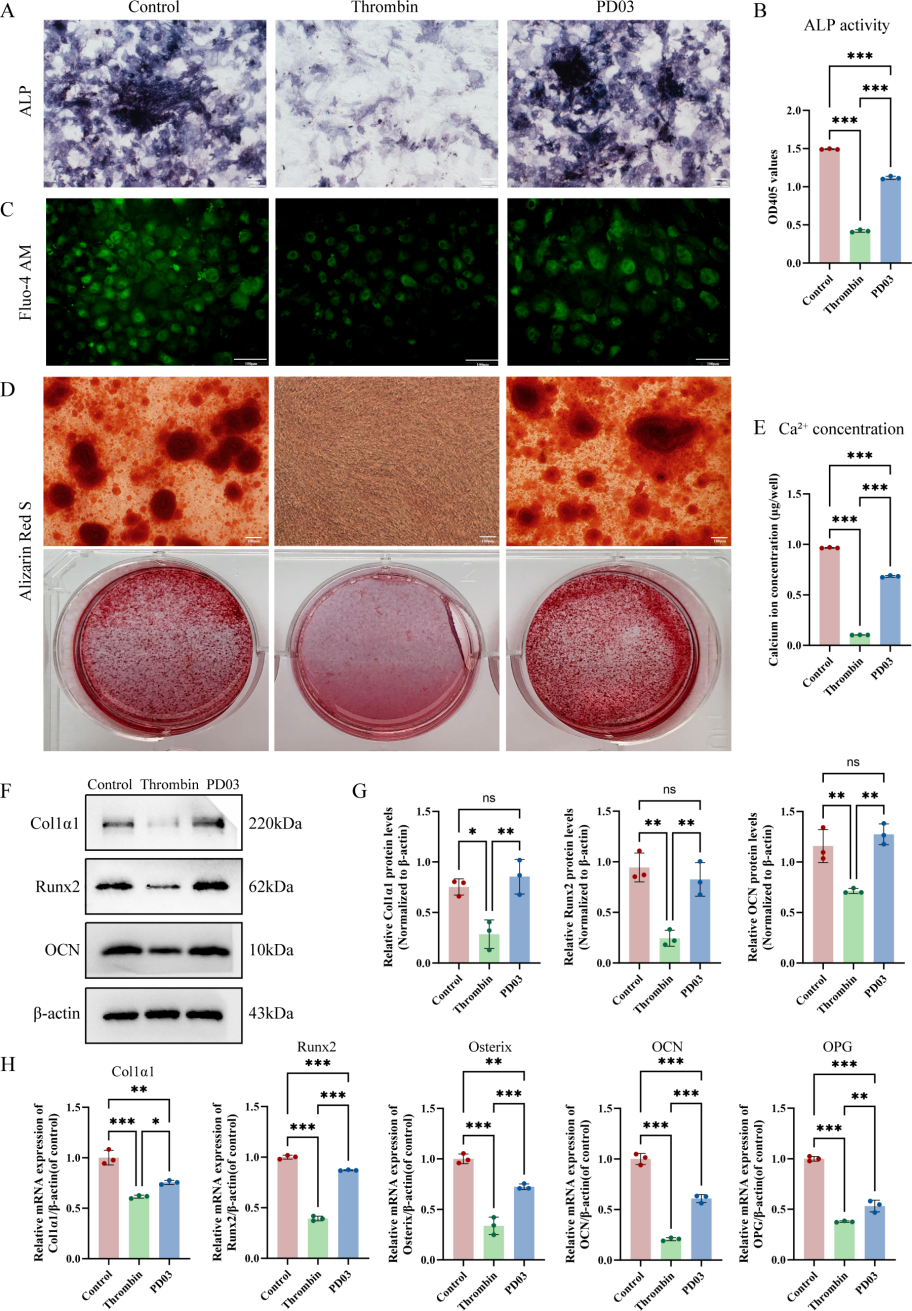
The expression levels of osteogenic differentiation markers after treatment with thrombin and PD03 **(A, B)** After 7 days of induction with osteogenic differentiation medium, ALP staining and quantitative analysis of ALP activity were conducted in osteoblasts. **(C)** Intracellular calcium levels were assessed using the calcium-sensitive probe Fluo-4 AM after treatment with the differentiation medium for 7 days. **(D, E)** Following 21 days of osteogenic induction, mineralized nodules were stained, and intracellular calcium concentration were quantified using a calcium assay kit. **(F)** Western blot analysis was used to examine the expression levels of Col1α1, Runx2 and OCN **(G)**The relative protein levels of Col1α1/β-actin, Runx2/β-actin and OCN/β-actin were measured using ImageJ software. **(H)** RT-qPCR analysis of osteogenic marker gene expression was performed relative to β-actin and normalized to the control group. Data are presented as mean ± SD (n = 3). P-values were determined by one-way ANOVA (multi-group comparisons) (*p < 0.05; **p < 0.01; ***p < 0.001; ns, P >0.05). Scale bar: 100 μm.

Consistent with its role as an early marker of osteogenic differentiation, ALP activity was significantly suppressed by thrombin treatment after being induced in osteogenic differentiation medium, as evidenced by both staining and quantitative assays. However, ALP levels were restored following PD03 treatment (**Figures 1A, B**). Loading cells with calcium-sensitive fluorescent probes (Fluo-4 AM) revealed a significant reduction in intracellular Ca²^+^ levels upon stimulation with thrombin, while PD03 treatment significantly enhanced the inhibitory effect of thrombin on calcium influx (**Figure 1C**). After 21 days of osteogenic induction, calcified nodule formation was evaluated by Alizarin Red-S staining, and quantitative analysis of intracellular calcium ions was conducted simultaneously. Thrombin treatment completely prevented the formation of calcified nodules and reduced intracellular calcium levels, and these inhibitory effects were reversed by PD03 (**Figures 1D, E**). In addition, western blot and qRT-PCR analysis demonstrated that PD03 significantly reversed the thrombin-mediated inhibition of osteogenic gene expression, including Col1α1, Runx2, Osterix, OCN, and OPG (**Figures 1F–H**).

### 3.2 PD03 suppressed thrombin-mediated osteoblast proliferation

**Figure 2:**
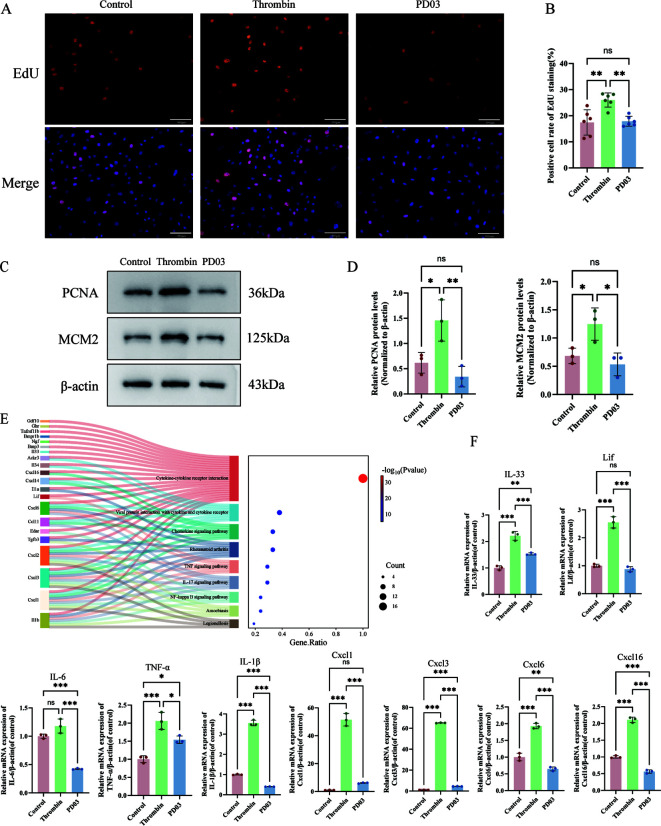
Regulation of osteoblast proliferation by thrombin and PD03. **(A)** The representative images for EdU incorporation assay results from thrombin- and PD03-treated osteoblasts for 24 hours. **(B)** The percentages of EdU-positive cells were analyzed using ImageJ software. **(C)** Western blot analysis was used to examine the expression levels of PCNA and MCM2 **(D)** The relative protein levels of PCNA/β-actin and MCM2/β-actin were measured using ImageJ software. **(E)** Bubble plot and Sankey diagram illustrating KEGG pathway enrichment for 21 DEGs related to inflammatory cytokines and the pathway cytokine-cytokine receptor interaction. **(F)** RT-qPCR analysis of mRNA levels of chemokines, inflammatory factors (represented by IL-1β, Cxcl1, and Cxcl3) in thrombin- and PD03-treated osteoblasts. Data are presented as mean ± SD (n = 3). P-values were determined by one-way ANOVA (multi-group comparisons) (*p < 0.05; **p < 0.01; ***p < 0.001; ns, P >0.05). Scale bar: 100 μm.

The proliferative effect of osteoblasts in the presence of thrombin and PD03 was assessed by EdU staining and the expression of proliferation-related proteins, such as PCNA and MCM2. The results showed that thrombin treatment significantly increased the number of EdU-positive osteoblasts (**Figures 2A, B**), accompanied by upregulated expression of proliferation-related proteins PCNA and MCM2 (**Figures 2C, D**). In contrast, PD03 intervention reduced the proportion of EdU-positive cells, as well as the expression of these proliferation markers.

The overlapping DEGs among the three groups were analyzed, revealing enrichment primarily in cytokine-related terms, which were visualized using a bubble plot and a Sankey diagram (**Figure 2E**). Subsequent experimental results demonstrated that PD03 significantly inhibited the upregulation of thrombin-induced inflammatory cytokines (IL-6, TNF-α, Lif, IL-1β, IL-33) and chemokines (Cxcl1, Cxcl3, Cxcl6, Cxcl16) (**Figure 2F**).

### 3.3 PD03 modulates the thrombin-mediated expression of cytokines, calcification- and extracellular matrix-regulatory genes in osteoblasts

**Figure 3:**
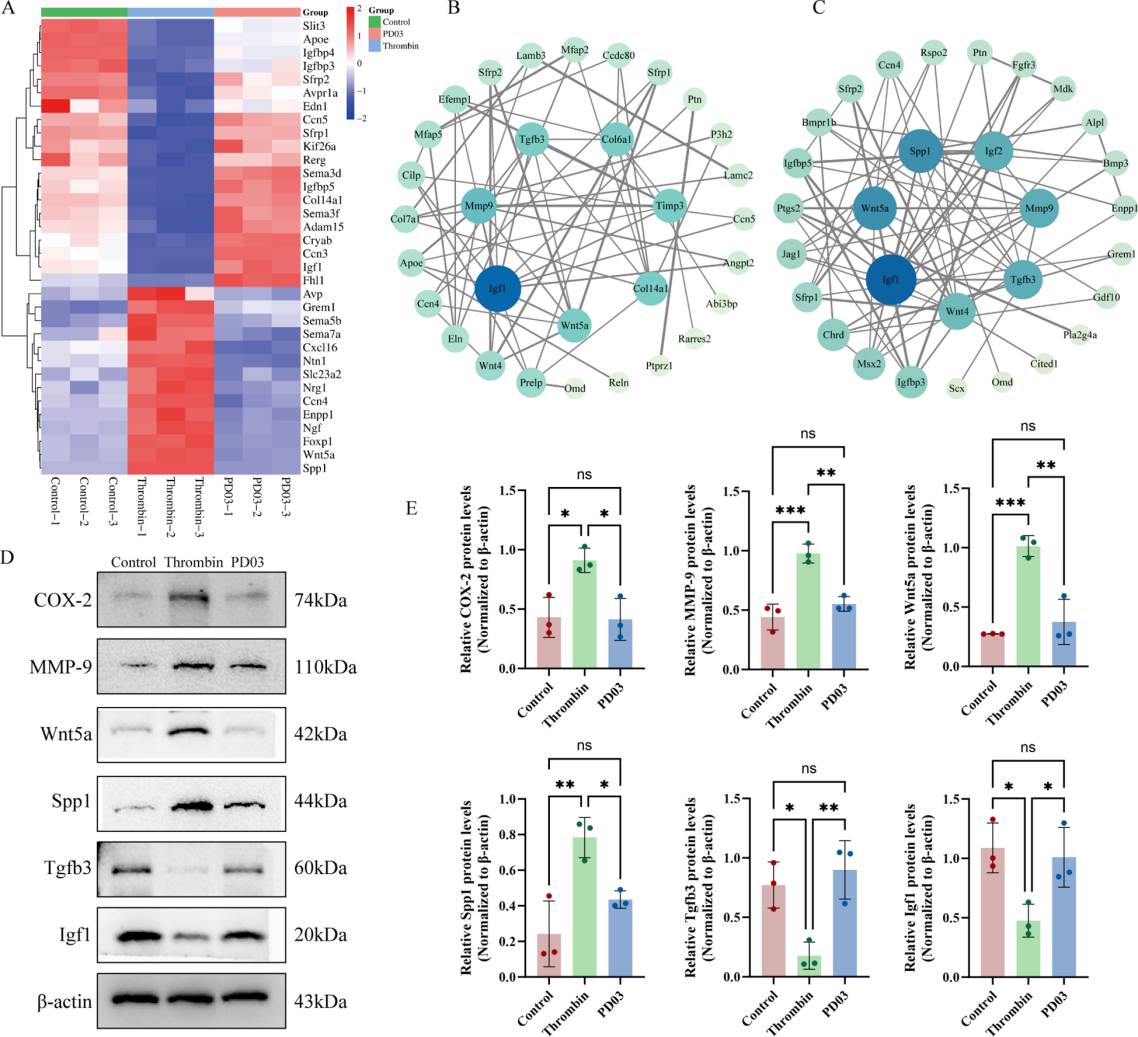
Experimental validation of bioinformatically identified inflammatory cytokines, ECM-related, and calcification-regulatory genes. **(A)** Heatmap illustrating genes involved in regulating osteoblast growth. **(B)** PPI network of calcification-regulatory genes from BP terms, highlighting hub genes such as Igf1, Tgfb3, and MMP-9. **(C)** PPI network of ECM-regulatory genes derived from CC terms, highlighting hub genes such as Igf1, Wnt5a, and Spp1. **(D)** Western blot analysis and quantitative analysis of COX-2/β-actin, MMP-9/β-actin, Wnt5a/β-actin, Spp1/β-actin, Tgfb3/β-actin and Igf1/β-actin ratios in thrombin- and PD03-treated osteoblasts. **(E)** The ratios of COX-2/β-actin, MMP-9/β-actin, Wnt5a/β-actin, Spp1/β-actin, Tgfb3/β-actin and Igf1/β-actin were compared among different groups. Data are presented as mean ± SD (n = 3). P-values were determined by one-way ANOVA (multi-group comparisons) (*p < 0.05; **p < 0.01; ***p < 0.001; ns, P >0.05). Scale bar: 100 μm.

Transcriptomic analysis during BP identified multiple genes regulating osteoblast growth (**Figure 3A**), among which Wnt5a and Igf1 are recognized for their critical roles in promoting osteoblast proliferation and differentiation (24, 25). PPI network analysis identified key ECM- and calcification-regulatory genes, demonstrating that hub genes such as Igf1, MMP-9, Wnt5a, and Tgfb3 are critically involved in these processes (**Figures 3B, C**). Subsequent experiments demonstrated that thrombin suppressed the expression of pro-osteogenic genes Igf1 and Tgfb3, while PD03 effectively reversed thrombin-induced downregulation of these genes (26, 27). Concurrently, thrombin treatment significantly upregulated COX-2, MMP-9, Wnt5a, and Spp1, with PD03 intervention attenuating thrombin’s regulatory effects on these gene expressions (**Figures 3D, E**).

### 3.4 Functional enrichment analysis of DEGs among the three groups, network pharmacology results, and molecular docking validation of the top 10 genes

**Figure 4:**
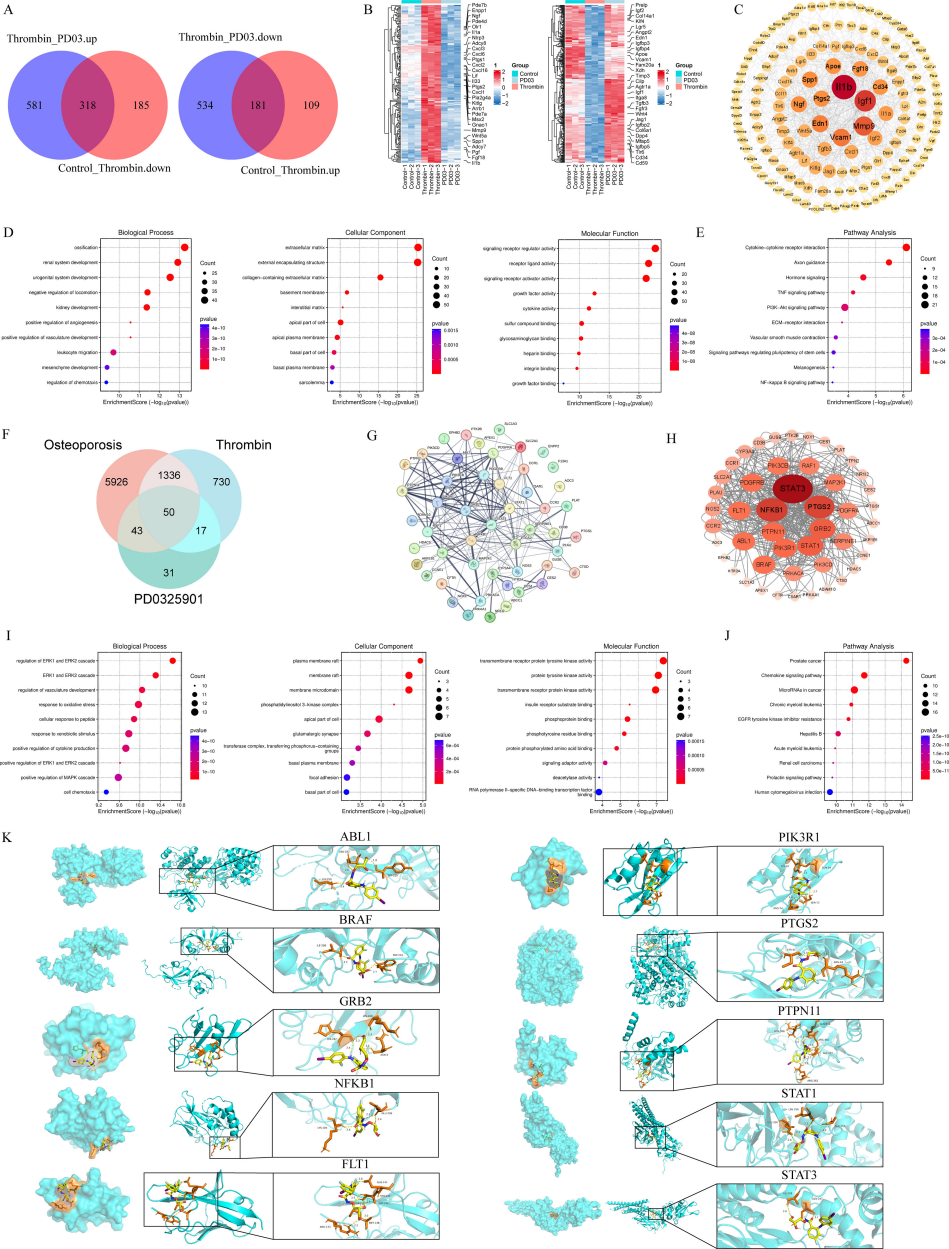
RNA-Seq, network pharmacology and molecular docking analysis. **(A)** The overlapping DEGs are displayed in Venn diagrams. **(B)** A heatmap was generated based on DEGs, with expression levels ranging from low (blue) to high (red). **(C)** Volcano plot analysis was performed to visualize the DEGs between control and thrombin groups, as well as between thrombin and PD03 groups, using significance thresholds of p < 0.05 and |log_2_FoldChange| > 0.58, with upregulated genes shown in orange and downregulated genes in green. **(D, E)** GO enrichment analysis was visualized using a bubble plot and included three categories: biological process (BP), cellular component (CC), and molecular function (MF), along with KEGG pathway analysis. **(F)** Venn diagram showing 50 overlapping targets among PD03, thrombin, and OP-predicted targets. **(G, H)** Protein-protein interaction (PPI) networks of the overlapping targets, generated via STRING and Cytoscape. **(I, J)** Bubble plots depicting the significance, enrichment, and gene count for key GO terms and KEGG pathways associated with the overlapping targets. **(K)** The left panel depicts the atomic binding interactions between PD03 and these core protein residues. The middle panel illustrates the overall binding conformation of the ligand-protein complex. The right panel provides an enlarged view of the binding site, with residue labels and yellow dashed lines denoting hydrogen bonds.

RNA-seq analysis identified 1908 DEGs among samples using a false discovery rate (FDR)-adjusted P < 0.05. The overlapping target genes were identified using Venn diagrams, revealing an overlap of 318 and 181 common genes among three datasets, respectively (**Figure 4A**). Transcript abundance was then visualized using a heatmap (**Figure 4B**). The PPI network constructed from transcriptomic DEGs revealed extensive interactions among the encoded proteins, with IL-1β, MMP-9, and Insulin-like Growth Factor-1(Igf1) exhibiting high connectivity and thus potentially serving as central hubs (**Figure 4C**). The GO analysis showed that biological processes (BP) were primarily enriched in calcification, cellular components (CC) in the ECM, and molecular functions (MF) in signaling receptor regulatory activity (**Figure 4D**). KEGG pathway enrichment analysis indicated that DEGs were predominantly associated with cytokine-cytokine receptor interaction. Additionally, the NF-κB signaling pathway was implicated in regulating DEG expression (**Figure 4E**).

The potential targets of PD03 were predicted using the databases SwissTargetPrediction. All targets were standardized using UniProt (https://www.uniprot.org/) to ensure consistency across the dataset. Osteoporosis-related targets were obtained from databases, such as GeneCards, using the keyword “osteoporosis”. Only targets with a relevance score above a predefined threshold (e.g., >10 in GeneCards) were retained. Thrombin-related targets were identified using GeneCards databases. A total of 50 overlapping targets among PD03-related, thrombin-related, and osteoporosis-related genes were visualized using Venn diagrams (**Figure 4F**) and further analyzed using the STRING website and Cytoscape (version 3.9.1) to construct protein-protein interaction (PPI) networks (**Figures 4G, H**). The significance, enrichment level, and number of DEGs associated with each GO term and KEGG pathway were visualized using bubble plots (**Figures 4I, J**).

Molecular docking was performed between PD03 and the top 10 core targets identified through network pharmacology. The results indicated that all selected target genes exhibited favorable binding affinities with PD03, with docking scores ranging from –5.2 to –8.8 kcal/mol (**Table 2**). Notably, PTGS2(COX-2) exhibits the strongest binding affinities, with docking scores below –8.8kcal/mol, indicating high binding stability (**Figure 4K**). Although NFKB1 (NF-κB) and STAT3 (Stat3) did not exhibit lower docking scores, previous studies have also confirmed their roles in osteogenic differentiation (28–31). Subsequent experiments will further validate the functions of COX-2, NF-κB and Stat3 in thrombin-regulated osteogenic differentiation.

**Table 2:** Molecular docking scores between PD03 and the top 10 genes from network pharmacology.

Potential targets	PDB ID	UniProt ID	Binding energy(kcal/mol)
ABL1	pdb_00007dt2	P00519	-6.4
BRAF	pdb_00003ny5	P15056	-7.9
GRB2	pdb_00003ove	P62993	-6.1
NFKB1	pdb_00008tqd	P19838	-5.9
FLT1	pdb_00005abd	P17948	-6.5
PIK3R1	pdb_00002iug	P27986	-6.4
PTGS2	pdb_00005f19	P35354	-8.8
PTPN11	pdb_00003b7o	Q06124	-6.9
STAT1	pdb_00008d3f	P42224	-5.2
STAT3	pdb_00006njs	P40763	-6.4

### 3.5 The crosstalk between the MEK-Erk1/2 and Stat3 signaling pathways in osteogenic differentiation

**Figure 5:**
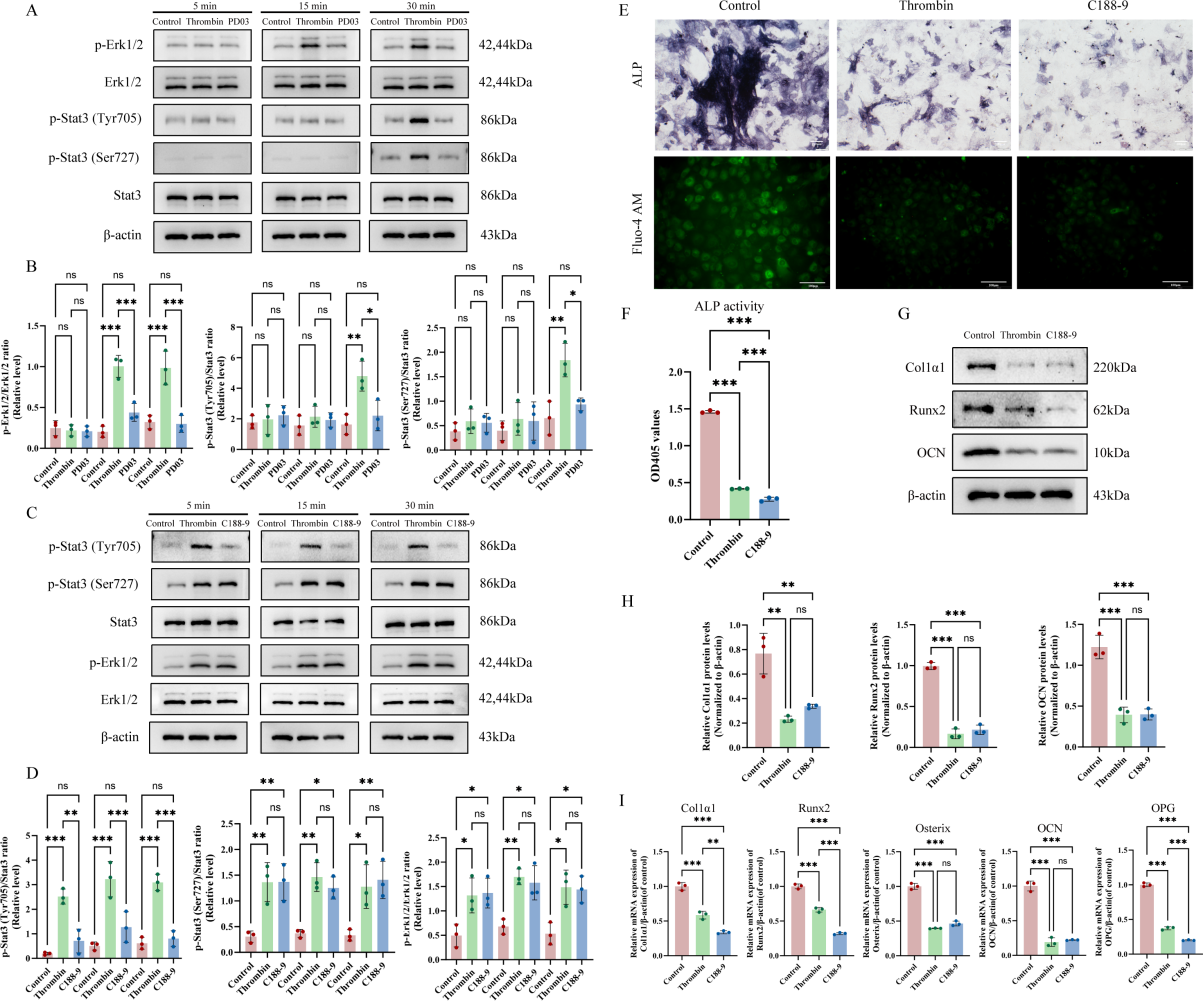
The mutual regulatory effects of MEK-Erk1/2 and Stat3 following treatment with PD03 and C188-9, and the role of Stat3 pathways in osteogenic differentiation. **(A, B)** The phosphorylation levels of Erk1/2, Stat3 (Y705), and Stat3 (S727) in osteoblasts were examined by western blot following treatment with thrombin and PD03 at different time points, and the relative protein levels of p-Erk1/2 to Erk1/2, p-Stat3(Y705) to Stat3, and p-Stat3(S727) to Stat3 were measured using Image J software. **(C, D)** The protein levels of p-Erk1/2, p-Stat3 (Y705), and p-Stat3 (S727) in osteoblasts were analyzed by western blot after being treated with thrombin and C188–9 at different time points, and the relative protein levels of p-Erk1/2 to Erk1/2, p-Stat3(Y705) to Stat3, and p-Stat3(S727) to Stat3 were evaluated using Image J software. **(E)** Osteoblasts were stained for ALP and Fluo-4 AM after being induced with the osteogenic differentiation medium for 7 days. **(F)** An enzymatic assay quantified ALP activities in cell lysates. **(G)** Western blot analysis was used to examine the expression levels of Col1α1, Runx2 and OCN. **(H)**The relative protein levels of Col1α1/β-actin, Runx2/β-actin and OCN/β-actin were measured using ImageJ software. **(I)** The expression levels of Col1α1, Runx2, Osterix, OPG, and OCN were assessed by qPCR following thrombin and C188–9 treatment for 7 days. Data are presented as mean ± SD (n = 3). P-values were determined by one-way ANOVA (multi-group comparisons) (*p < 0.05; **p < 0.01; ***p < 0.001; ns, P >0.05). Scale bar: 100 μm.

Emerging evidence suggests a complex crosstalk between the MEK-Erk1/2 and Stat3 signaling pathways that regulate cellular inflammation and differentiation (32, 33). The phosphorylation levels of Erk1/2 in osteoblasts were markedly increased following thrombin treatment for 15 and 30 minutes (mins), whereas phosphorylation of Stat3 (S727 and Y705) was upregulated only at 30 minutes, and PD03 treatment attenuated the expression of these phosphorylated proteins correspondingly, the ratios of p-Erk1/2 to Erk1/2 is statistically significant in the thrombin group vs the control group or the PD03 group at 15 and 30mins (**Figures 5A, B**). Furthermore, treatment of osteoblasts with C188–9 for 5, 15, and 30 minutes failed to suppress thrombin-induced phosphorylation of Erk1/2, with no statistically significant changes observed in the relative protein levels of p-Erk1/2 to Erk1/2 (**Figures 5C, D**). After one week of induced osteogenic differentiation, C188–9 slightly aggravated the thrombin-mediated suppressive effects on osteogenic differentiation, as evidenced by ALP staining and activity **[****Figure 5E, F** (upper panel)], the accumulation of Ca^2+^ inside the cytosol (**Figures 5E**, lower panel), and both the protein and mRNA expression levels of osteogenic genes. (**Figures 5G–I**).

### 3.6 The mutual regulatory relationship between thrombin-activated MEK-Erk1/2 and NF-κB pathways during the process of osteoblast differentiation

**Figure 6:**
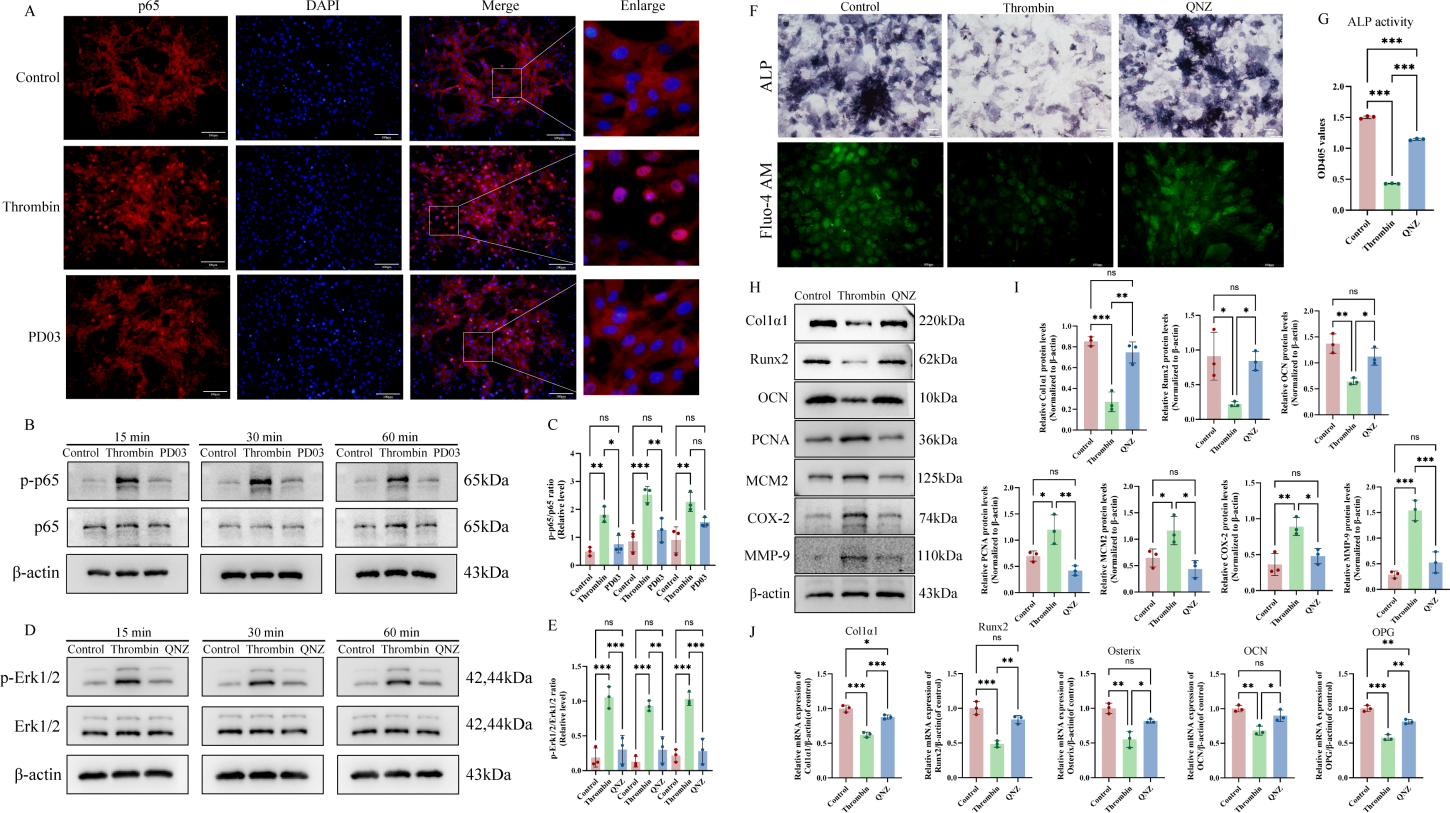
Regulatory relationships between NF-κB and MEK-Erk1/2 pathways in thrombin-induced osteoblast differentiation. **(A)** Nuclear translocation of p65 in osteoblasts was assessed by immunofluorescence. **(B)** Phosphorylated levels of p65 in thrombin and PD03-treated osteoblasts at 15min, 30min, and 60min were determined by western blot. **(C)** Relative protein levels of p-p65 to p65 were quantified using ImageJ software. **(D)** Phosphorylated levels of Erk1/2 in osteoblasts following treatment with thrombin and QNZ for 15min, 30min, and 60min were assessed by western blot. **(E)** Relative protein levels of p-Erk1/2 to Erk1/2 were quantified using ImageJ software. **(F)** ALP staining (upper panel) and intracellular calcium signaling (lower panel) were evaluated by the test kits. **(G)** ALP activities in thrombin- and QNZ-treated osteoblasts for 7 days were measured by colorimetric assay. **(H)** Expression of osteogenic marker genes (Col1α1, Runx2, and OCN), proliferation-related genes (MCM2, PCNA) and hub genes (MMP-9, COX-2) in thrombin- and QNZ-treated osteoblasts was evaluated by western blot. **(I)** Relative protein levels of Col1α1/β-actin, Runx2/β-actin, OCN/β-actin, MCM2/β-actin, PCNA/β-actin, MMP-9/β-actin, and COX-2/β-actin were quantified using ImageJ software. **(J)** qRT-PCR was used to analyze the expression of osteogenic marker genes (e.g., Runx2, Osterix, and OCN) in thrombin- and QNZ-treated osteoblasts for 7 days. Data are presented as mean ± SD (n = 3). P-values were determined by one-way ANOVA (multi-group comparisons) (*p < 0.05; **p < 0.01; ***p < 0.001; ns, P >0.05). Scale bar: 100 μm.

**Figure 7:**
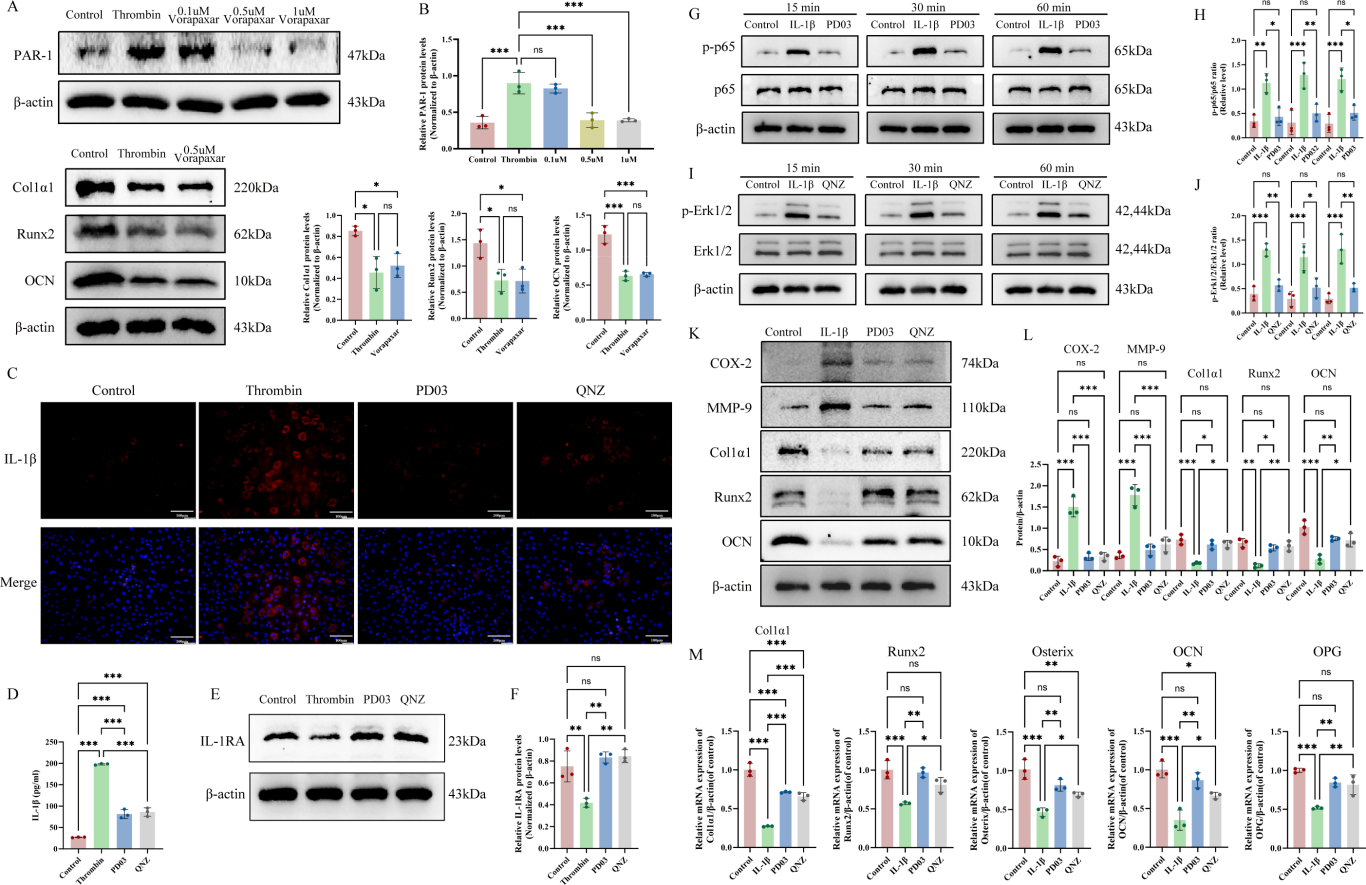
The modulation of PD03 on osteoblast differentiation mediated by the IL-1β-activated feedback loop between NF-κB and MEK-Erk1/2 pathways. **(A)** PAR-1, Col1α1, Runx2 and OCN protein levels in osteoblasts were assessed by western blot analysis. **(B)** The quantitative analysis of PAR-1, Col1α1, Runx2 and OCN expression. **(C)** Thrombin-stimulated IL-1β expression in osteoblasts was inhibited by PD03 and QNZ, as determined by immunofluorescence. **(D)** PD03 and QNZ inhibited thrombin-stimulated IL-1β secretion in osteoblasts, as measured by ELISA. **(E)** IL-1RA protein levels in osteoblasts were assessed by western blot analysis. **(F)** The quantitative analysis of IL-1RA expression. **(G)** PD03-mediated modulation of NF-κB signaling pathways activated by IL-1β in osteoblasts. **(H)** Relative protein levels of p-p65 to total p65 in osteoblasts were quantified using ImageJ software. **(I)** The regulatory effect of QNZ on the IL-1β-activated MEK-Erk1/2 signaling pathway in osteoblasts. **(J)** Relative protein levels of p-Erk1/2 to total Erk1/2 in osteoblasts were quantified using ImageJ software. **(K)** The regulatory effects of PD03 and QNZ on IL-1β-induced COX-2, MMP-9, Col1α1, Runx2 and OCN expression were evaluated by western blot analysis. **(L)** The ratios of MMP-9/β-actin, COX-2/β-actin, Col1α1/β-actin, Runx2/β-actin and OCN/β-actin were compared among different groups. **(M)** The expression of osteogenic markers (e.g., Runx2, Osterix, Col1a1, OPG, and OCN) was evaluated by RT-qPCR. Data are presented as mean ± SD (n = 3). P-values were determined by one-way ANOVA (multi-group comparisons) (*p < 0.05; **p < 0.01; ***p < 0.001; ns, P >0.05). Scale bar: 100 μm.

Network pharmacology analysis identified NF-κB as a core gene in the “thrombin–osteoporosis-PD03” axis. We further investigate the regulatory relationship between NF-κB and MEK-Erk1/2 signaling pathways. Following the inhibition of the MEK-Erk1/2 pathway (PD03 group), thrombin-induced nuclear translocation of p65 was markedly reduced, and the phosphorylation level of p65 was also significantly suppressed (**Figures 6A, B**), and the ratio of p-p65/p65 is statistically significant in the thrombin group vs the control group or the PD03 group (p < 0.05) (**Figure 6C**). Upon inhibition of NF-κB signaling, thrombin-induced Erk1/2 phosphorylation was substantially attenuated (**Figure 6D****),** and the ratio of p-Erk1/2/Erk1/2 is statistically significant in the thrombin group vs the control group or the QNZ group (p < 0.05) (**Figure 6E**). In parallel, osteogenic parameters were also impaired in the thrombin-treated group, as evidenced by decreased ALP staining and activity **[****Figures 6F, G** (upper panel)], and reduced intracellular calcium signaling **[****Figures 6F**, lower panel], attenuated expression of osteogenic marker genes, and upregulated proliferation-related genes (MCM2 and PCNA) as well as sequencing-derived hub genes (MMP-9 and COX-2**) (****Figure 6H**). The relative protein levels of proliferation-related genes and hub genes identified in **Figure 7H** exhibited statistically significant differences in the thrombin-treated group compared to both the control and QNZ-treated groups (p < 0.05) (**Figure 6I**). In addition, qRT-PCR analysis demonstrated that QNZ significantly reversed the thrombin-mediated inhibition of osteogenic gene expression, including Col1α1, Runx2, Osterix, OCN, and OPG (**Figure 6J**).

### 3.7 Regulatory effects of PD03 on thrombin-induced IL-1β-mediated osteogenic differentiation via modulation of MEK-Erk1/2 and NF-κB pathways in osteoblasts

To verify whether thrombin inhibits osteogenic differentiation via protease-activated receptor-1 (PAR-1), a primary thrombin receptor involved in diverse cellular responses, osteoblasts were treated with thrombin in the presence of Vorapaxar, and the expression of key osteogenic markers was evaluated. The findings showed that Vorapaxar failed to reverse thrombin’s inhibitory effect on osteogenic differentiation, confirming that this process is independent of the thrombin/PAR-1 signaling pathway (**Figures 7A, B**).

Based on sequencing results, PPI analysis indicated that IL-1β was highly central within the regulatory network and identified as a pivotal hub gene. The results of both IF and ELISA demonstrated that thrombin significantly induced IL-1β expression and mature IL-1β secretion in osteoblasts, which were efficiently inhibited by PD03 and QNZ (**Figures 7C, D**). To further assess the IL-1R signaling activation, the expression of the endogenous IL-1 receptor antagonist (IL-1RA) was examined. Thrombin markedly reduced IL-1RA levels, suggesting that it exerts a bidirectional regulatory effect on inflammation by activating IL-1R signaling by disrupting the balance between IL-1β and its endogenous antagonist IL-1RA. Notably, PD03 and QNZ restored IL-1RA expression, highlighting their upstream regulatory effects (**Figures 7E, F**). Subsequent experiments showed that PD03 (a MEK inhibitor) potently suppressed IL-1β-induced activation of the NF-κB signaling pathway (**Figure 7G**). In contrast, QNZ (an NF-κB inhibitor) effectively blocked IL-1β-induced MEK-Erk1/2 pathway activation (**Figure 7I**). The ratios of phosphorylated Erk1/2 to total Erk1/2 and phosphorylated p65 to total p65 are significantly elevated in the IL-1β-treated group compared to the control, PD03-treated, or QNZ-treated groups (p < 0.05) (**Figures 7H, J**). Moreover, inhibition of the MEK-Erk1/2 pathway (by PD03) and the NF-κB pathway (by QNZ) restored the expression of osteogenesis-related markers suppressed by IL-1β, an effect potentially associated with increased expression of COX-2 and MMP-9 (**Figure 7K****),** with the ratios of COX-2/β-actin, MMP-9/β-actin, Col1α1/β-actin, Runx2/β-actin and OCN/β-actin significantly upregulated in the IL-1β group compared to the PD03 and QNZ groups (p < 0.05) (**Figure 7L**). Concurrently, qRT-PCR analysis demonstrated that inhibition of these two pathways restored IL-1β-suppressed osteogenesis-related markers, including Col1α1, Runx2, Osterix, OCN, and OPG (**Figure 7M**).

### 3.8 The effects of MMP-9 knockdown and COX-2 overexpression in osteoblasts on osteogenic differentiation

**Figure 8:**
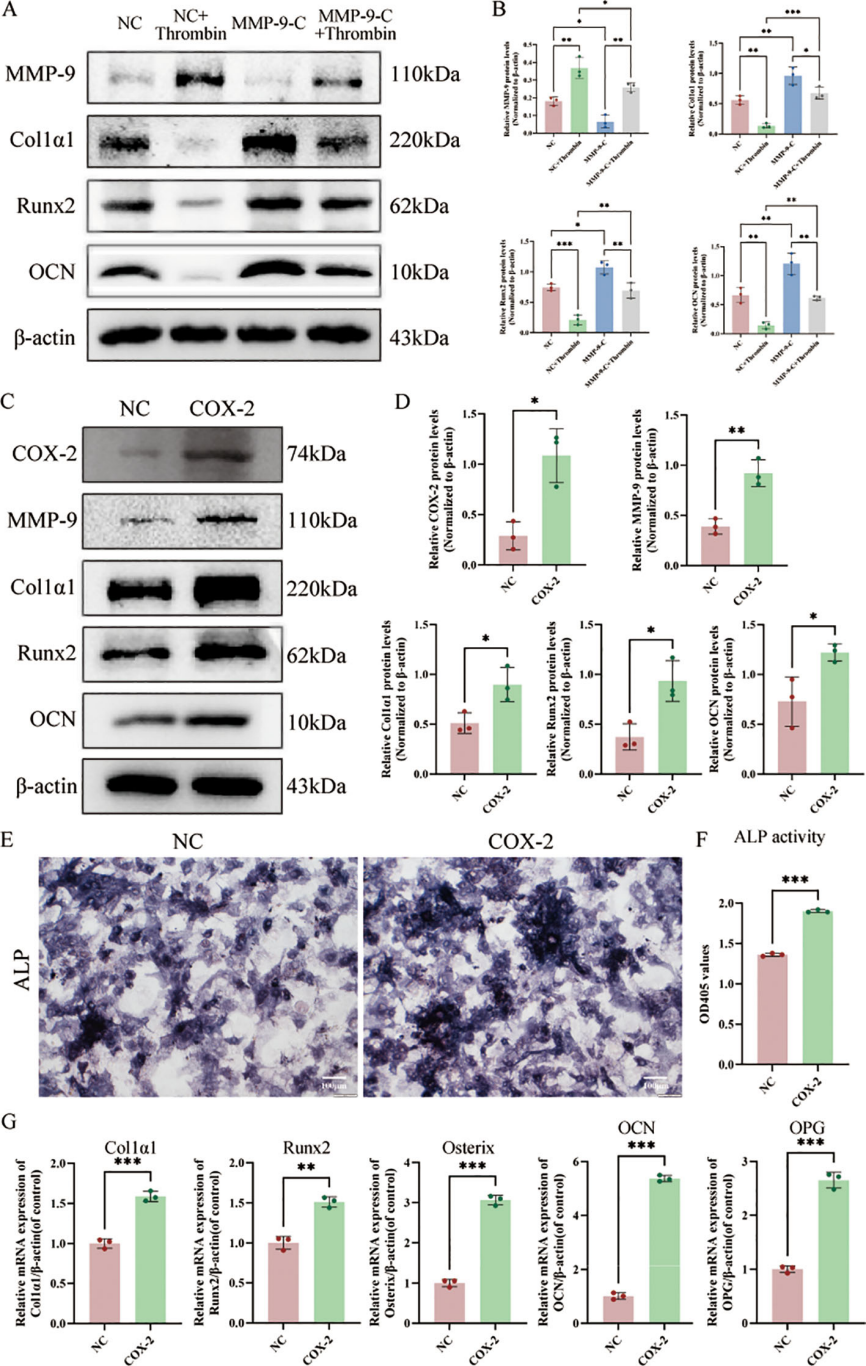
Knocking down MMP-9 promotes osteogenic differentiation, whereas overexpression of COX-2 slightly enhances osteogenic differentiation. **(A)** MMP-9, Col1α1, Runx2 and OCN protein levels in osteoblasts following MMP-9 knockdown were assessed by western blot analysis. **(B)** The quantitative analysis of MMP-9, Col1α1, Runx2 and OCN expression. **(C)** COX-2, MMP-9, Col1α1, Runx2 and OCN protein levels in osteoblasts following COX-2 overexpression were assessed by western blot. **(D)** The quantitative analysis of COX-2, MMP-9, Col1α1, Runx2 and OCN expression. **(E)** Representative images of ALP staining at day 7 post-transfection. **(F)** Quantitative ALP activity assay in the COX-2 group relative to NC. **(G)** qRT-PCR analysis of osteogenic markers (Col1a1, Runx2, Osterix, OCN, and OPG), and comparison between the COX-2 group and the NC group. Data are presented as mean ± SD (n = 3). P-values were determined by Student’s t-test (two-group comparisons) (*p < 0.05; **p < 0.01; ***p < 0.001; ns, P >0.05). Scale bar: 100 μm.

Three siRNAs were designed and synthesized to target MMP-9 (**Supplementary Table 1**). Based on knockdown efficacy evaluated by western blot, MMP-9-C siRNA was selected for subsequent experiments (**Supplementary Figure 3**). To investigate the role of MMP-9 in osteoblast differentiation, osteoblasts were transfected with either MMP-9-C siRNA (MMP-9-C group) or a negative control siRNA (NC group). Transfection of osteoblasts with MMP-9 siRNA markedly reduced MMP-9 protein levels and upregulated the expression of osteogenic marker genes (Col1α1, Runx2, and OCN), even in the presence of thrombin, effectively reversing thrombin-induced suppression of osteogenic differentiation (**Figures 8A, B**).

To investigate the role of COX-2 in osteoblast differentiation, osteoblasts were transfected with both COX-2 overexpressing plasmid (COX-2 group) and control plasmid (NC group). Western blot analysis confirmed that transfection with the COX-2 overexpression plasmid significantly elevated COX-2 and MMP-9 protein levels compared to endogenous expression (**Figure 8C**), with the ratios of COX-2/β-actin and MMP-9/β-actin upregulated in the COX-2 group compared to the NC group (p < 0.05) (**Figure 8D**). ALP staining revealed enhanced enzymatic activity in the COX-2-overexpressing group compared to controls. Quantitative analysis confirmed a significant elevation in ALP activity in the COX-2 group compared to the NC group (**Figures 8E, F**). Overexpression of COX-2 in osteoblasts resulted in a significant upregulation of osteogenic markers, including Col1a1, Runx2, Osterix, OCN, and OPG (**Figure 8G**).

## 4 Discussion

The pathological hallmark of OP is the imbalance between bone resorption and bone formation, with a particular emphasis on the dysregulation between osteoblasts and osteoclasts, which plays a central role in the disease progression (34). Excessive osteoclast activity and insufficient osteoblast function are the main mechanisms underlying OP, and current research primarily focuses on the functional regulation of these two cell types.

In many diseases, insufficient thrombin can lead to bleeding. However, in numerous conditions, elevated thrombin levels not only contribute to thrombosis but may also exert profound effects on other physiological systems. Thrombin significantly inhibits RANKL/M-CSF-induced osteoclastogenesis in mouse bone marrow and human peripheral blood mononuclear cells (PBMCs) (35). Currently, the role of thrombin in osteogenic differentiation remains inconclusive.

The increase in thrombin levels is observed in various autoimmune diseases that are also associated with higher risks of fractures and OP. For example, thrombin promotes synovial hyperplasia in rheumatoid arthritis (RA), which is a hallmark of RA pathology. The proliferative and invasive behavior of fibroblast-like synoviocytes not only sustains inflammation but also mediates cartilage degradation and bone erosion, contributing to the irreversible joint destruction observed in RA (36–38).

Patients with inflammatory myopathies exhibit a markedly enhanced thrombin generation potential, along with a higher prevalence of OP and an increased risk of fractures, indicating a co-existing prothrombotic and bone-compromised state (39, 40). A similar phenomenon is observed in multiple sclerosis, and patients with systemic sclerosis (SSc) are also prone to reduced bone mineral density and fragility fractures (41–43).

Given the increased thrombin generation potential observed in patients with chronic inflammatory diseases accompanied by OP, and the identification of “calcification” as the most significantly enriched biological process term in the analysis of DEGs, a process closely associated with osteoblast differentiation, it is essential to investigate whether thrombin directly influences osteogenic differentiation. Exploring its regulatory effects on bone-forming cells may uncover novel mechanisms underlying inflammation-associated bone loss. This may provide a theoretical basis and therapeutic strategies for managing OP and fractures caused by elevated thrombin levels.

The transition from pre-osteoblasts to mature bone-forming cells typically involves three stages, each characterized by specific markers. The proliferation phase is primarily characterized by the expression of PCNA and MCM2, along with low levels of Runx2 and ALP. The ECM secretion and maturation phase is marked by the initial expression of osteogenic genes such as Runx2, Osterix/Sp7, ALP, Osteopontin (OPN), and Collagen Type I (Col1a1). During this stage, cells begin to secrete ECM components and gradually transition toward mineralization. The matrix mineralization phase is characterized by the deposition of calcium salts into the ECM, leading to the formation of mineralized nodules. This phase is primarily characterized by the expression of late-stage osteocyte marker genes Osteocalcin (OCN) (44).

Theoretically, thrombin promotes osteoblast proliferation by significantly increasing the number of EdU-positive osteoblasts, accompanied by elevated expression of PCNA and MCM2, thereby enhancing osteoblast differentiation correspondingly. However, thrombin significantly attenuated ALP activity and downregulated the expression of multiple osteogenic genes following 7 days of osteogenic induction. Furthermore, after 21 days of induction, thrombin markedly inhibited mineralized nodule formation, as well as reduced intracellular calcium concentrations and the fluorescence intensity of calcium-sensitive probes in osteoblasts.

KEGG enrichment analysis of both the sequencing data and network pharmacology revealed significant enrichment of cytokine-related signaling pathways. Further analysis of inflammatory mediator expression within these KEGG-enriched pathways revealed that thrombin significantly upregulated the expression of CXCL family members (Cxcl1, Cxcl3, Cxcl6, and Cxcl16), along with classical proinflammatory cytokines such as TNF-α, IL-1β, IL-6, and Lif, and these results of bioinformatics analysis were subsequently confirmed through experimental validation.

IL-1β, also identified as one of the hub genes in the PPI network analysis based on DEGs, along with other genes such as MMP-9 and Igf1, plays a pivotal regulatory role in the osteogenic process, a conclusion further supported by previous studies. For instance, conditional deletion of the Igf1 gene in osteocytes resulted in impaired bone formation and resorption, altered skeletal morphology and mineralization, as well as reduced bone length in mice (45). Overexpression of Igf1 restores the proliferative and osteogenic differentiation capacity of senescent bone marrow-derived MSCs (46). The serum levels of MMP-9 in osteoporotic patients were significantly higher than those in non-osteoporotic individuals (47). Additionally, animal experiments further confirmed the negative correlation between MMP-9 levels and BMD (bone mineral density), suggesting the potential role of elevated MMP−9 as an early biomarker for OP (48). Our experiments further revealed that thrombin suppressed Igf1 expression and upregulated MMP-9 expression, both of which were involved in modulating ECM synthesis and calcification in osteoblasts, indicating a potential inhibitory role of thrombin in osteogenic differentiation. PD03 intervention can inhibit the osteoblastic proliferation, upregulate the expression of osteogenic marker genes, calcification-regulatory genes, and ECM regulatory genes in the presence of thrombin.

Network pharmacology analysis revealed significant enrichment of BP terms within the Erk1/2 signaling cascade, primarily driven by protein tyrosine kinase (PTK) activity, as confirmed by MF enrichment analysis. It has been previously reported that IL-1β-stimulated hyperactivation of the MEK−Erk1/2 signaling pathway inhibits osteoblast differentiation (10). PPI network analysis further identified Stat3 and NF-κB as central regulatory hubs, implicating the involvement of the Stat3 and NF-κB pathways in the mechanism of action of thrombin-OP-PD03. Most studies currently support that activation of the Stat3 pathway promotes the osteogenic differentiation of preosteoblasts. Further study also confirmed that the absence of Stat3 in osteoblasts can lead to a decrease in bone formation rate in mice, resulting in craniofacial deformities, OP, and spontaneous fractures (28). Cytokines from the IL-6 family (e.g., IL-6 and Lif) are key regulators of this process by inducing Stat3 phosphorylation. Sustained activation of Stat3 enhances osteogenesis, whereas genetic deletion of Stat3 in osteoblasts leads to impaired bone formation (29).

To further investigate the potential crosstalk between these two pathways, both the MEK inhibitor PD03 and the Stat3-specific inhibitor C188–9 were applied to stimulate osteoblasts. Thrombin treatment activated both the Erk1/2 and Stat3 signaling pathways in osteoblasts. Inhibition of the MEK-Erk1/2 pathway with PD03 reduces Erk1/2 phosphorylation, thereby decreasing the thrombin-induced expression of IL-6, a key activator of Stat3, suggesting that MEK-Erk1/2 inhibition indirectly modulates Stat3 activity by altering cytokine production. However, C188–9 failed to inhibit Erk1/2 signaling. Strikingly, thrombin stimulation for 15 and 30 minutes significantly increased Erk1/2 phosphorylation, and Stat3 phosphorylation was absent at 15 minutes but became evident at 30 minutes. These findings further suggest that the MEK-Erk1/2 pathway may function upstream of Stat3 to regulate its activation. Although activation of the IL-6 (Lif)/Stat3 signaling pathway is generally reported to promote osteogenic differentiation, our experimental results showed that thrombin activated this pathway while simultaneously inhibiting osteogenesis. This discrepancy may be attributed to the activation of inflammatory pathways or factors that negatively regulate osteogenic differentiation.

To rule out the participation of thrombin in inhibiting osteogenic differentiation via the thrombin/PAR-1 pathway, the PAR-1 antagonist Vorapaxar was used. The results showed that Vorapaxar did not alter thrombin’s inhibitory effect on osteogenic marker gene expression, strongly indicating that thrombin suppresses osteogenic differentiation independently of the conventional PAR-1 signaling pathway. This finding suggests the presence of alternative or parallel signaling mechanisms.

Previous studies have also demonstrated that the activation of the NF-κB pathway or IL-1β can inhibit osteoblast differentiation (30, 31). Consistently, PPI analysis based on DEGs and network pharmacology also identified IL-1β, NF-κB, and COX-2 as crucial hub genes in osteogenic differentiation. To further investigate whether thrombin regulates osteogenic differentiation through IL-1β-mediated modulation of the MEK-Erk1/2 and NF-κB pathways, we examined the effects of IL-1β and thrombin on the activity of these two pathways and osteogenic differentiation. Selective inhibition of the thrombin- or IL-1β-activated MEK-Erk1/2 and NF-κB signaling pathways uncovered reciprocal regulation, with each pathway capable of both activating and suppressing the other, thereby establishing a positive feedback loop. Moreover, inhibition of the NF-κB pathway, which is activated by thrombin and IL-1β, produced effects on osteogenic gene expression, ALP activity, and cell proliferation that were consistent with those observed following MEK-Erk1/2 inhibition.

To confirm the role of IL-1β in this feedback loop, we assessed its intracellular expression by immunofluorescence and measured secreted mature IL-1β in culture supernatants using ELISA. Thrombin treatment significantly increased IL-1β expression. Furthermore, to comprehensively assess the regulatory role of thrombin on the IL-1R signaling pathway, the expression of the endogenous IL-1 receptor antagonist (IL-1RA) was also assessed. Interestingly, thrombin treatment significantly reduced IL-1RA expression. Both PD03 and QNZ efficiently restored the expression levels of IL-1β and IL-1RA. Collectively, these data indicate that thrombin plays a dual role in the IL-1R signaling pathway by regulating upstream molecules in the NF-κB and MEK-Erk1/2 pathways, such as IL-1β and IL-1RA.

Notably, PPI analyses based on DEGs and network pharmacology both indicated a vital role of COX-2. In our experiments, inhibition of either the MEK-Erk1/2 pathway or the NF-κB pathway attenuated the thrombin-induced upregulation of both MMP-9 and COX-2, suggesting that these genes function downstream of both signaling pathways. Moreover, the negative regulatory role of MMP-9 in osteogenic differentiation has been well established (47, 48). To further confirm the function of MMP9 in thrombin-treated osteoblastic differentiation, MMP-9 knockdown indeed promoted osteogenic differentiation. COX-2 plays a dual role in the osteogenic differentiation of MSCs. A previous study indicates that reduced COX-2 expression in MSCs enhances osteogenic gene expression and mineralization (49). Another study demonstrates that COX-2 deficiency significantly impairs osteoblastogenesis (50). Concurrently, studies have demonstrated that localized overexpression of COX-2 promotes osteoblast differentiation (51). Therefore, to verify its role in thrombin-related osteogenic differentiation, COX-2 was overexpressed in osteoblasts. The results indicate that COX-2 overexpression in osteoblasts promotes osteogenic differentiation, suggesting that the inhibition of osteogenic differentiation by thrombin-induced activation of the MEK-Erk1/2 and NF-κB pathways is not mediated through increased COX-2 expression. In other words, among the downstream genes of the MEK-Erk1/2 and NF-κB pathways, there are both osteogenesis-promoting and osteogenesis-inhibiting genes, but the inhibitory genes predominate in function.

Research on the role of MEK-Erk1/2 in osteoblast differentiation remains controversial. For instance, Xiao et al. reported that activation of MEK-Erk1/2 by tensile loading promotes osteoblast differentiation, which differs from our findings. This discrepancy may be attributed to differences in stimuli: thrombin triggers the release of numerous inflammatory cytokines in osteoblasts, thereby suppressing their differentiation, a mechanism consistent with inflammation-induced bone loss. This study reinforces the pivotal role of thrombin as an inflammatory factor (33).

**Figure 9:**
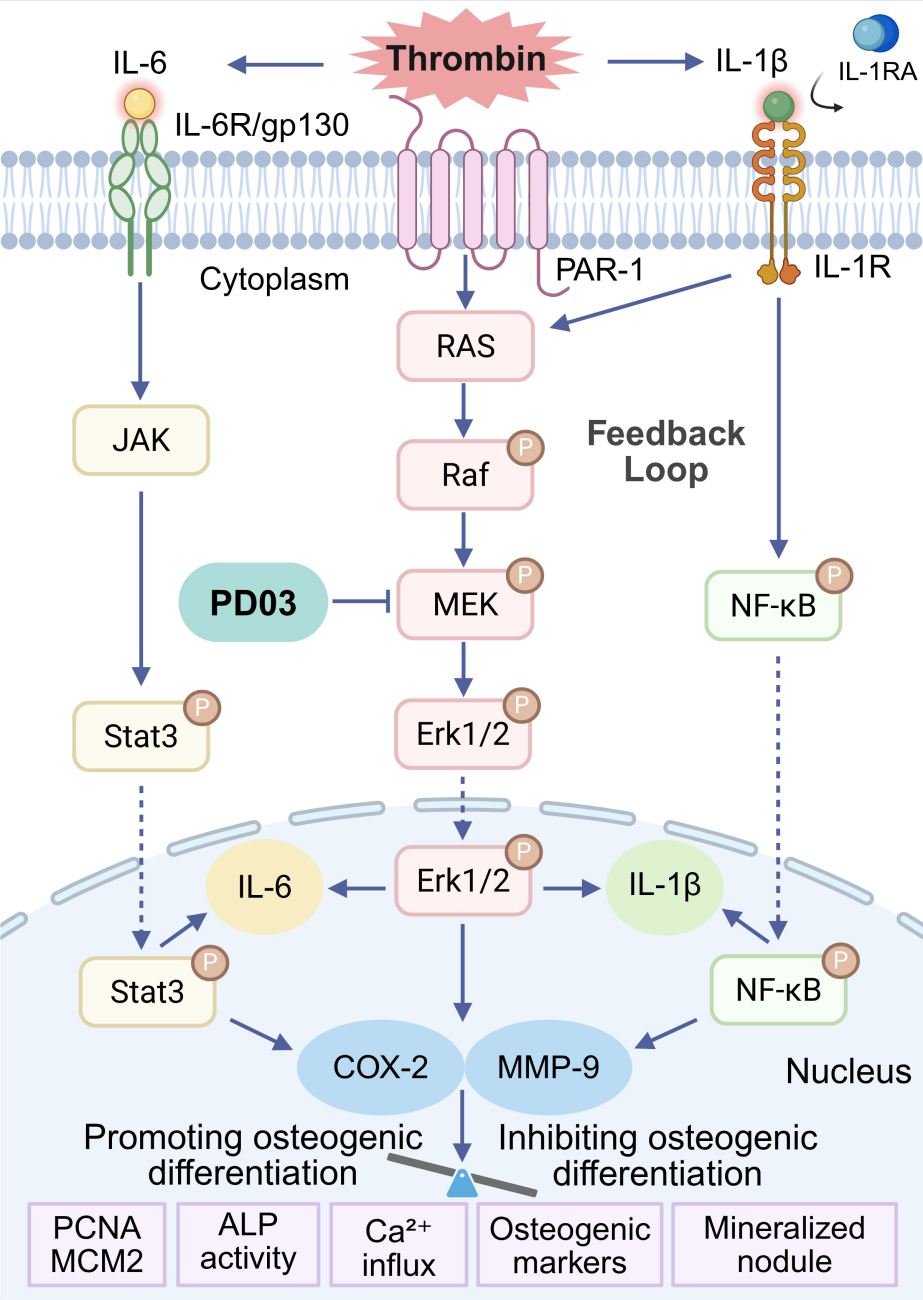
Diagram of mechanism. Thrombin inhibits osteogenic differentiation by activating the IL-1β-mediated feedback loop between the MEK-Erk1/2 and NF-κB signaling pathways. This figure was created with BioRender.com.

In this study, we integrated the advantages of different omics approaches. For instance, transcriptomics reflects gene expression at the mRNA level, while network pharmacology predicts drug targets at the protein level. Ultimately, we demonstrated that thrombin inhibits osteogenic differentiation, likely by upregulating classical proinflammatory cytokines such as IL-1β, which in turn activates the MEK-Erk1/2 and NF-κB pathways. This hypothesis provides a novel perspective on the molecular mechanisms linking thrombin to the pathogenesis of OP. These two pathways form a feedback loop that further regulates downstream molecules such as MMP-9. The schematic representation of this mechanism is illustrated in **Figure 9**. Remarkably, both transcriptomics and network pharmacology consistently suggested an important regulatory role of COX-2. However, transient overexpression of COX-2 in osteoblasts slightly promoted osteogenic differentiation, indicating that the pro-osteogenic effect of thrombin-induced COX-2 expression is negligible in this study.

Although our study demonstrates that PD03 can reverse thrombin-induced inhibition of osteogenic differentiation, its development as a potential therapeutic agent for osteoporosis remains challenging. PD03 is a selective MEK inhibitor, currently investigated primarily in preclinical or early-phase clinical studies for conditions such as tumors (52, 53). Systemic administration may carry off-target effects and dose-limiting toxicities, including dermatologic reactions, ocular toxicity, and gastrointestinal disturbances (54). Moreover, the MEK-Erk1/2 signaling pathway plays a role in multiple physiological processes, and the long-term consequences of its inhibition on skeletal homeostasis remain unclear. While the concentration of PD03 used in this study (0.1 μM) effectively reversed thrombin-induced inflammatory and osteogenic-inhibitory phenotypes *in vitro*, its accumulation in bone tissue and long-term safety require further evaluation in animal models. Therefore, we consider that the therapeutic potential of PD03 should be comprehensively assessed through additional preclinical studies that address pharmacodynamics, pharmacokinetics, and toxicology. Future strategies, such as local delivery or bone-targeted approaches, may improve its translational feasibility.

## 5 Conclusion

Using RNA-seq analysis, network pharmacology, and experimental validation, this study confirmed that thrombin suppresses osteoblast differentiation via an IL-1β-mediated feedback loop between the MEK-Erk1/2 and NF-κB signaling pathways, establishing PD03 as a potential therapeutic agent for OP.

## Data Availability

The data presented in the study are deposited in the NCBI repository, accession number GSE320308.
