# Supplementary figures and images for: PD0325901 alleviates thrombin-inhibited osteogenic differentiation through an IL-1β-activated feedback loop between MEK-Erk1/2 and NF-κB signal pathways: insights from bioinformatics and experimental verification
Source: Front Immunol. 2026 Mar 9;17:1730337. doi: 10.3389/fimmu.2026.1730337 (PMC13006212; doi:10.3389/fimmu.2026.1730337)

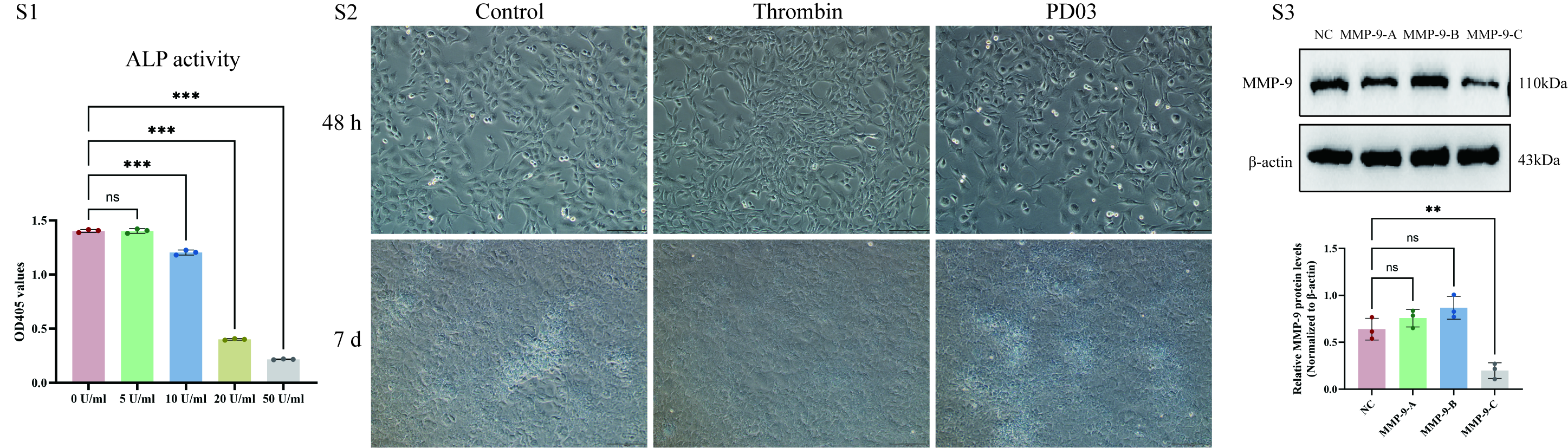

Supplement: Supplementary file 1 [file Image1.tif]
