# Supplementary material for: PD0325901 alleviates thrombin-inhibited osteogenic differentiation through an IL-1β-activated feedback loop between MEK-Erk1/2 and NF-κB signal pathways: insights from bioinformatics and experimental verification
Source: Front Immunol. 2026 Mar 9;17:1730337. doi: 10.3389/fimmu.2026.1730337 (PMC13006212; doi:10.3389/fimmu.2026.1730337)
Supplement: Supplementary file 2 [file Table1.docx]

**Supplementary Table 1.** Sequences of the siRNA

| **Name** | **Sense (5’-3’)** | **Anti-sense (5’-3’)** |
| --- | --- | --- |
| MMP-9-A | GGUGCGCGACGACGUCGAA | UUCGACGUCGUCGCGCACC |
| MMP-9-B | CCUAUUGGAUCCAAAGCUA | UAGCUUUGGAUCCAAUAGG |
| MMP-9-C | GGCUCUAGGCUACAGCUUU | AAAGCUGUAGCCUAGAGCC |
